# Audit feedback interventions to address high-risk prescriptions in long-term care homes: a costing study and return on investment analysis

**DOI:** 10.1186/s43058-021-00225-7

**Published:** 2021-10-28

**Authors:** Kednapa Thavorn, Srishti Kumar, Catherine Reis, Jonathan Lam, Gail Dobell, Cara Mulhall, Jeremy M. Grimshaw, Noah Ivers

**Affiliations:** 1 Clinical Epidemiology Program, Ottawa Hospital Research Institute, Ottawa, Ontario Canada; 2 School of Epidemiology and Public Health, University of Ottawa, Ottawa, Ontario Canada; 3 ICES, Toronto, Ontario Canada; 4 Women’s College Institute for Health System Solutions and Virtual Care, Toronto, Ontario Canada; 5 Ontario Health (Quality), Toronto, Ontario Canada; 6 Institute for Health Policy, Management and Evaluation, University of Toronto, Toronto, Ontario Canada

**Keywords:** Audit and feedback, Antipsychotics prescribing, Cost analysis, Return on investment

## Abstract

**Background:**

Audit and feedback is a common implementation strategy, but few studies describe its costs. ‘MyPractice’ is a province-wide audit and feedback initiative to improve prescribing in nursing homes. This study sought to estimate the costs of ‘MyPractice’ and assess whether the financial benefit of ‘MyPractice’ offsets those costs.

**Methods:**

We conducted a costing study from the perspective of the Ontario government. Total cost of ‘MyPractice’ was calculated as the sum of the costs of producing and disseminating the reports (covering three report releases) which were obtained from Ontario Health staff interviews and document reviews. Return on investment (ROI) was calculated as the ratio of net cost-savings and the intervention cost. Cost savings were based on the effectiveness of ‘MyPractice’ derived from a published cohort study. Cost-savings attributable to ‘MyPractice’ were estimated from the changes in the rates of antipsychotics over time between physicians who signed up and viewed the reports and those who did not sign up to the reports.

**Results:**

Total intervention costs were C$223,691 (C$838 per physician and C$74,564 per release). Costs incurred during the development phase accounted for 74% of the total cost (C$166,117), while implementation costs for three report releases were responsible for 26% of the total costs (C$57,575). The ROI for every C$1 spent on the ‘MyPractice’ intervention was 1.02 (95% CI 0.51, 1.93) for three report releases.

**Conclusion:**

‘MyPractice’ report offers a good return on investment and the value for money could improve with greater number of report releases.

**Supplementary Information:**

The online version contains supplementary material available at 10.1186/s43058-021-00225-7.

Contributions to literature

- The present study evaluates the cost and return on investment for an audit and feedback initiative, namely ‘MyPractice’, that addresses prescribing of high-risk medications among long-term care home residents in Ontario, Canada.
- Our study shows that ‘MyPractice’ offers a good return on investment, and the value for money could improve with greater number of report releases.
- Findings could help inform the decision to adopt or expand such an audit and feedback initiative to address prescribing of high risk medications in long term care homes.

## Background

Approximately 20.7% of long-term care home residents are prescribed antipsychotics without concurrent psychiatric diagnosis in Canada [1]. Inappropriate use of these medications could increase risk of stroke, heart disease, and kidney failures which increase risk of premature death [1, 2]. To reduce inappropriate prescribing of antipsychotics among long-term care home residents, quality improvement initiatives are increasingly being utilized [3]. Audit and feedback (A&F) has emerged as a common method to improve the quality of health care practices. A&F interventions highlight discrepancies between desired and actual performance and encourage prescribers to address these discrepancies [4].

Previous studies have shown the effectiveness of A&F on prescriber behaviors [5–7]. An Australian study, for example, examining the impact of A&F on antipsychotics prescribing for schizophrenia showed that A&F was effective in changing prescribing rates towards recommended levels [6]. Another randomized control trial reported that the multi-strategic intervention consisting of A&F, including staff education and interdisciplinary reviews, resulted in a significant reduction in the proportion of long-term care home residents taking antipsychotics [7].

Despite growing evidence on effectiveness of A&F, little is known about the resources required to develop and implement such intervention. Given the constraint on health care budgets, credible information about A&F intervention costs could help inform the decision on whether an A&F intervention should be adopted or expanded. In this study, we estimated the costs of the ‘MyPractice’ reports, the A&F intervention that targets prescribing of antipsychotic medications in long-term care homes in Ontario and assessed whether the added costs could offset the benefits gained from the intervention.

## Methods

### Setting and intervention

Prescription drug costs for long-term care home residents in Ontario are covered by the Ontario Drug Benefit Program. Ontario Health, formerly Health Quality Ontario (HQO), is the agency that advises government and health care providers in Ontario on evidence to support high-quality care‚ to support improvements in quality, and to monitor and report on quality of health care provided in Ontario. The agency produces several ‘MyPractice’ clinician-focused reports with input from relevant stakeholders, including clinicians, epidemiologists, quality improvement experts, front-line clinicians, health services researchers, sector organizations and associations, and policy makers (4). The report aimed to address high-risk prescriptions for long-term care home residents in the province of Ontario, Canada, with an initial focus on antipsychotic medications. The reports were promoted to all family physicians working in long-term care homes across the province via communication materials distributed by the agency and external partners. The reports provided physicians the opportunity to voluntarily sign up to receive quarterly confidential feedback reports which compare the recipient’s prescribing rate with the provincial average. Physicians were asked to provide consent to receive the ‘MyPractice’ reports on the HQO website and verify their email address and identity. Physicians who signed up received an email notification when the report became available for download. They could log into their account through HQO’s secure web portal, download and view the report. Several audit and feedback initiatives are available to family physicians in Ontario; however, most physicians, especially those working in nursing homes, do not engage in these initiatives [8, 9].

The study period started from April 2016 to June 2017, covering development and implementation phases of ‘MyPractice’ reports focusing on antipsychotics. The ‘MyPractice’ initiative was designed and developed over 8 months (from April to November 2016). Three ‘MyPractice’ reports were released over a period of 7 months (from December 2016 to June 2017). At the time of the study, 267 physicians (28%) of 944 eligible physicians working in long-term care homes across Ontario signed up to receive the reports [4].

### Study design

We conducted a costing study of the ‘MyPractice’ intervention from the perspective of government of Ontario (i.e., the insurer). We also calculated the return on investment (ROI) for ‘MyPractice’ by comparing the intervention cost to savings from the reduction in antipsychotic medication prescriptions, as a result of the intervention. The ROI analysis is a method to estimate net financial gains (or losses) from an intervention, considering resources invested to implement the program and the amount gained though increase in revenue, reduced costs, or both [10]. The target population for ROI analysis included all physicians working in long-term care homes in Ontario who were provided the opportunity to sign up to receive ‘MyPractice’ reports.

### Resource use data collection and unit cost

We estimated the intervention cost using a gross costing technique, where costs were calculated as a product of resource use and unit costs. We obtained resource use data for developing and implementing the intervention and their unit costs from program financial records, service level agreements, and the program budget, through close consultation with Ontario Health staff. Costs were categorized into development and implementation costs.

Development costs comprised of the costs of data acquisition and personnel time for planning, report content development, developing technical infrastructure and administrative, and managerial support for the development phase. Implementation costs included the costs of personnel time for analysis, quality assurance, outreach, support for participant queries, administrative and managerial support. Cost data were presented in 2019 Canadian dollars.

### Analysis

#### Cost analysis

We estimated the total intervention cost as the sum of development and implementation costs for three report releases. We calculated the cost per physician by dividing the total intervention costs by the number of physicians who worked in long-term care homes and signed up to receive ‘MyPractice’ reports. We also projected the cost per physician if the ‘MyPractice’ initiative was adopted as a mandatory program, i.e., all physicians working in long-term care homes in Ontario received ‘MyPractice’ reports. Furthermore, we calculated the intervention cost per release by dividing the total intervention costs by the total number of report releases. Key drivers of development and implementation costs were also described.

In addition, we forecasted the annual cost of the ‘MyPractice’ intervention for the first year and subsequent years. The annual cost of ‘MyPractice’ for the first year was calculated as the sum of development costs and implementation costs for four quarterly report releases, while the upfront development costs were excluded for each subsequent year.

#### ROI analysis

The ROI of the ‘MyPractice’ intervention was calculated as [10]:

$$ \frac{\mathrm{Cost}\kern0.17em \mathrm{Savings}\hbox{-} \mathrm{Intervention}\kern0.17em \mathrm{Cost}}{\mathrm{Intervention}\kern0.17em \mathrm{Cost}} $$

A ROI greater than zero indicates that the savings generated from the intervention are greater than the costs of the developing and implementing the intervention.

Cost savings due to ‘MyPractice’ were based on the attributable effectiveness of ‘MyPractice’, which was measured as the change in antipsychotic prescription rates over time between the intervention and reference groups. For our base-case analysis, the intervention group comprised of physicians who signed-up and viewed the reports, while physicians who did not sign-up were considered the reference group. In a scenario analysis, physicians who signed up and did not view the report were considered the reference group.

Cost-savings were estimated from a retrospective cohort study that compared the mean proportion of long-term care home days on antipsychotics over the quarter before ‘MyPractice’ reports were released, the quarter immediately following the first report and the quarter that followed [11]. The cohort study reported changes in prescription rates over 6 months between the three exposure groups after adjustment for nursing home, physician, and resident characteristics [11]. Baseline prescription rates for antipsychotics were similar across the three exposure groups (signed up and viewed: 25.0%; signed up and not viewed: 26.3%; not signed up: 25.8%). For physicians who signed up and viewed the report, there was a 0.94% decrease in the mean proportion of long-term care home days on antipsychotics (95% CI 0.35%, 1.54%) compared to those who did not the sign up [11], and a 0.47% decrease (95% CI − 0.15%, 1.09%) compared to those who signed up and did not view the report.

Based on the reduction in the mean proportion of days on antipsychotics reported in the cohort study, we estimated cost-savings attributable to ‘MyPractice’ by multiplying the antipsychotic medication cost saved per resident and the total number of long-term care home residents in the province of Ontario [11]. Antipsychotic medication cost saved per resident was estimated by multiplying the reduction in the number of days on antipsychotics attributable to ‘MyPractice’ [11] and the daily costs of antipsychotic medications. Daily costs of antipsychotic medications were calculated by multiplying the prevalence of atypical and typical antipsychotic drugs commonly prescribed for nursing home residents with their daily costs [12, 13].

We performed scenario analyses projecting the ROI for ‘MyPractice’ covering four and eight report releases, with the assumptions that reports were released every quarter and that the effectiveness for ‘MyPractice’ reports observed over the study period was sustained for subsequent report releases.

We performed a probabilistic sensitivity analysis using the Monte Carlo simulation technique. We varied all parameters over ± 25% of their base values and repeated the ROI analysis over 1,000 iterations. Of these 1000 iterations, we estimated the probability that that ‘MyPractice’ would provide a good return on investment (i.e., ROI ≥ 0).

## Results

### Cost analysis

The total intervention costs were estimated to be C$223,691. The cost per physician who signed up for ‘MyPractice’ reports was C$838. The cost per release was C$74,564. The cost of developing the intervention was C$166,117 (74% of total cost) and the cost of implementation for three report releases was C$57,575 (26% of total costs). Data acquisition and analysis accounted for the largest share (35%) of development costs, followed by report production (29%) and management (29%) costs (Table 1). Quality assurance of reports accounted for majority (55%) of implementation costs. If the ‘MyPractice’ initiative was mandatory, the cost per physician was projected to be C$237.

**Table 1:** Breakdown of development and implementation costs

	Description	Cost (C$)	% of sub-total
**Development phase**			
Report production	Content development	46179	27.80%
	Editing	1827	1.10%
Technical infrastructure	Development of feedback survey	2062	1.24%
	Technical development of web portal	6987	4.21%
Data acquisition and analysis	Indicator development and third-party vendor costs for data	58613	35.28%
Administrative support	Scheduling of meetings	3197	1.92%
Management	Resource, partnership, and relationship management	47252	28.45%
***Total***		***166116***	***100.00%***
**Implementation phase (3 report releases)**			
Data acquisition and analysis	Third party vendor costs for data	8971	15.58%
Fact checking and quality assurance	Ensuring report accuracy	31767	55.18%
Project management coordination support	Support (online- and telephone-based) for queries by participants and timeline revisions	3447	5.99%
Outreach	Generating FAQs and website pop up feature	6569	11.41%
Administrative support	Scheduling of meetings	1776	3.08%
Management	Resource, partnership, and relationship management	5045	8.76%
***Total***		***57575***	***100.00%***

*Abbreviations*: *A&F* audit feedback, *FAQs* frequently asked questions

**Fig. 1:**
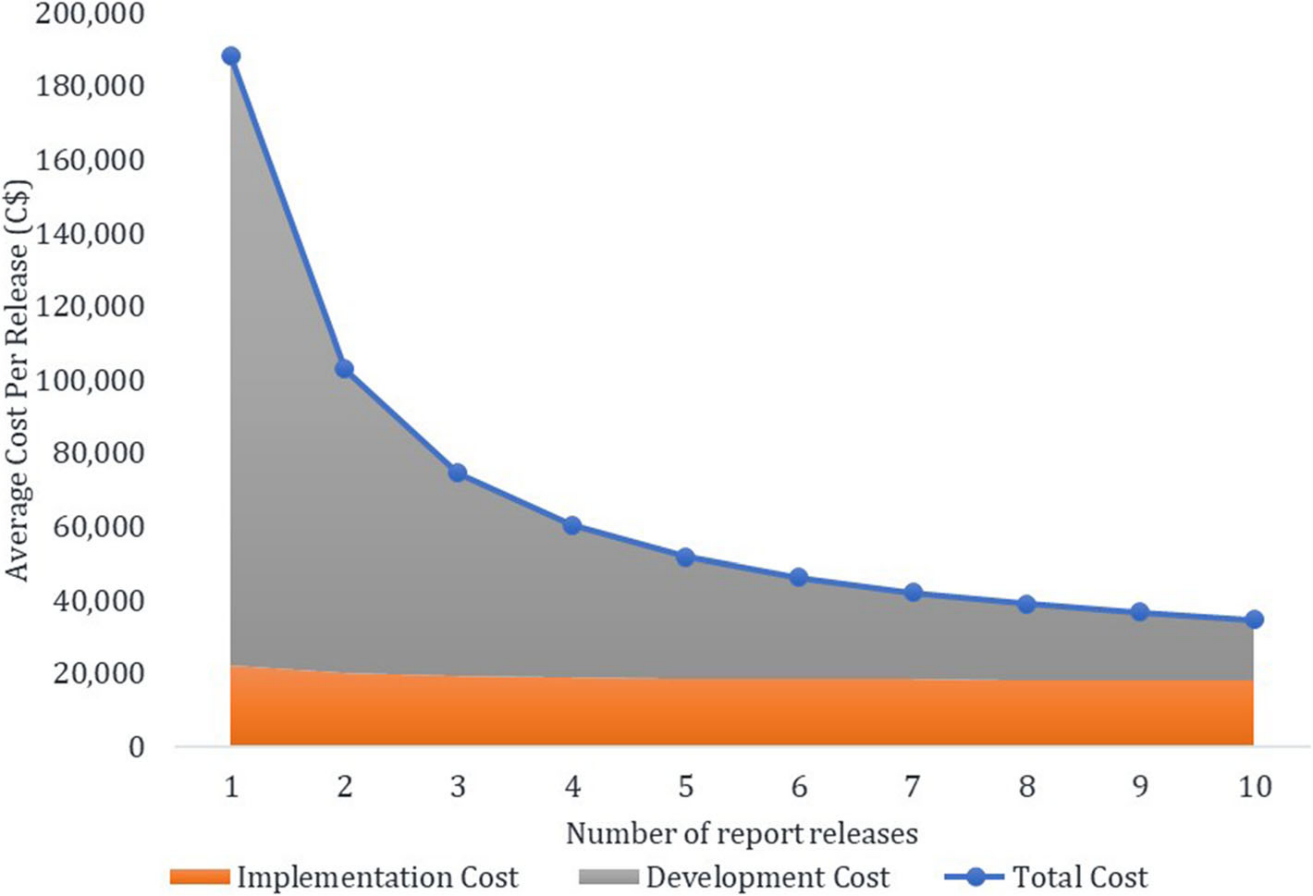
Average cost of ‘MyPractice’ by the number of report releases

Since most costs incurred upfront, the average cost per release decreased significantly with increasing number of report release (Fig. 1). We forecasted that if ‘MyPractice’ were to be released quarterly, its annual cost was estimated to be C$241,370 for the first year (December 2016 to December 2017) and C$70,716 for each subsequent year.

### ROI analysis

**Fig. 2:**
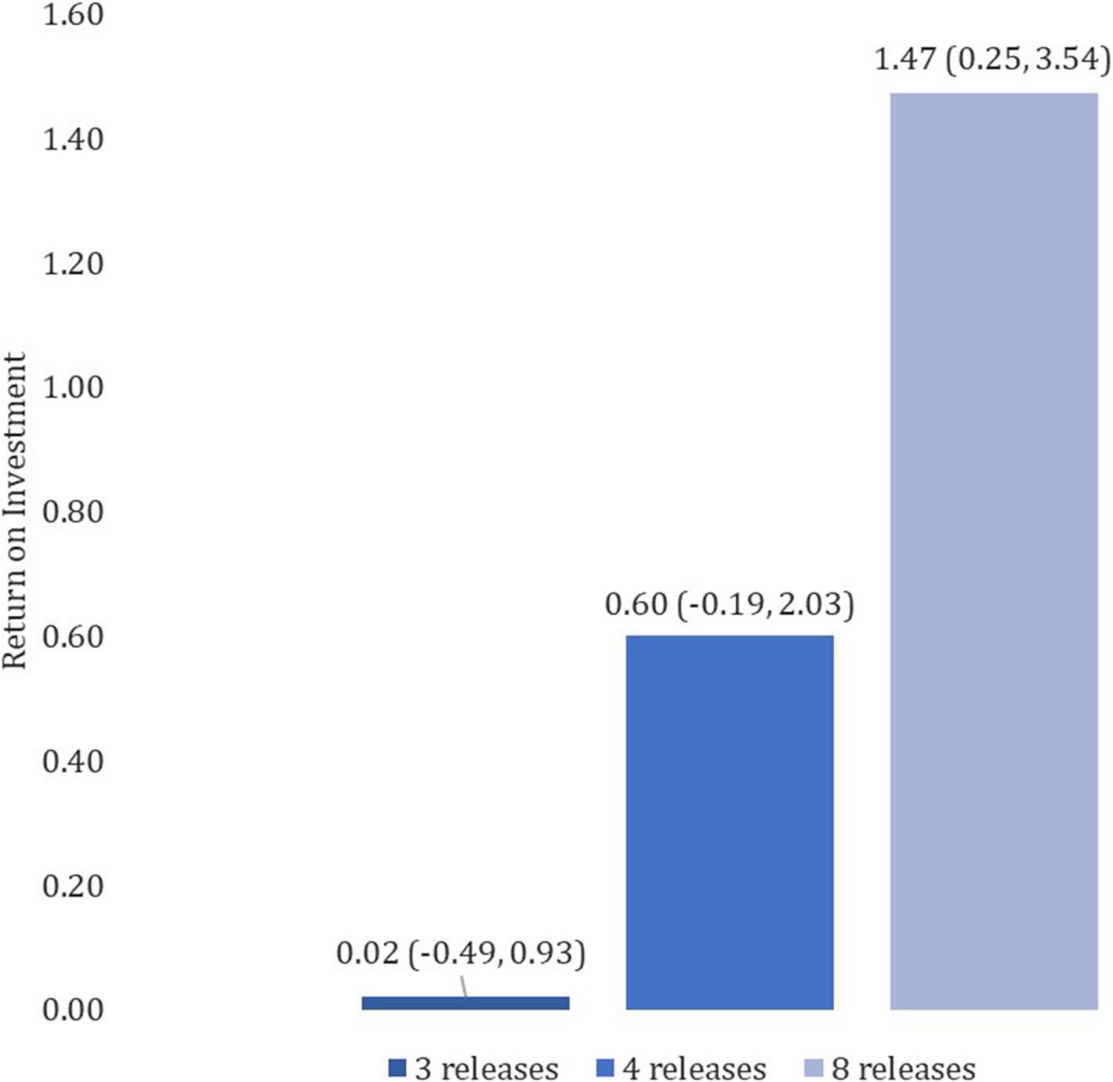
ROI for ‘MyPractice’ by the number of releases

The ROI for ‘MyPractice’ for three report releases was 0.02 (95% CI − 0.49, 0.93) for the base-case. Furthermore, the ROI increased with greater number of report releases (Fig. 2). The projected ROI was 0.60 (95% CI − 0.19, 2.03) for four releases and 1.47 (95% CI 0.25, 3.54) for eight releases.

Results from the scenario analysis showed that the ROI was highly sensitive to the effectiveness of ‘MyPractice’. The larger impact of the intervention on the mean proportion of long-term care home days on antipsychotics, the greater the ROI values. The ROI for ‘MyPractice’ was − 0.51 (95% CI − 0.93, 1.14) in a scenario analysis where the attributable effectiveness of ‘MyPractice’ was calculated using the physicians who signed up and did not view the reports as a reference group.

**Fig. 3:**
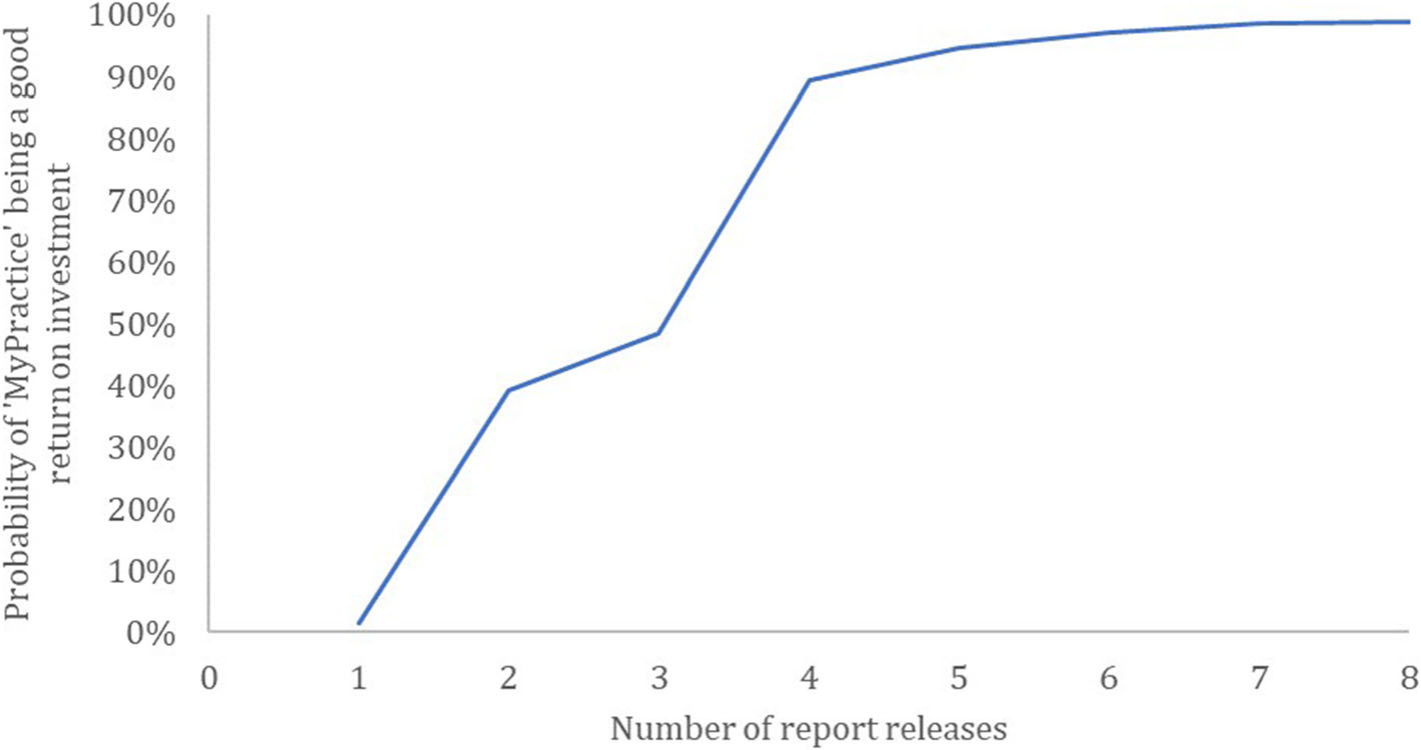
Return on investment acceptability curves by the number of report releases

The probability of ‘MyPractice’ being a good return on investment (ROI ≥ 0) was 48.5% for the base-case; this probability reached 89.3% if at least four reports were released (Fig. 3).

## Discussion

Our study highlights that the majority of ‘MyPractice’ costs (74%) were fixed and incurred upfront; the average cost per release‚ therefore‚ reduced significantly with increasing number of releases. This would suggest that releasing more reports or scaling the intervention to larger number of long-term care homes would not significantly increase the total costs of the intervention. We observed a positive ROI (ROI = 0.02) for the ‘MyPractice’ initiative, suggesting that for every C$1 spent on ‘MyPractice’, C$1.02 will be gained through the reduction in costs associated with antipsychotic prescriptions. The ROI was found to increase with the greater number of report releases if the same reduction in antipsychotic prescribing attributable to ‘MyPractice’ was sustained over subsequent report releases. The ROI was highly sensitive to the attributable reduction in antipsychotic prescription rates as a result of ‘MyPractice’ reports.

We identified only one existing study that assessed the costs of implementing an A&F intervention. Fretheim et al. determined the costs of implementing a multifaceted intervention consisting of audit and feedback, outreach visits and computerized reminders to improve adherence to clinical practice guidelines for prescribing of antihypertensive and cholesterol-lowering drugs in primary practices in Norway [14]. The intervention was conducted across 70 primary practices over 1 year. The total intervention cost for 257 physicians was C$122,584^1^ (C$476.98 per physician). The total intervention cost included the costs of training, software development, printing, salary of pharmacists doing outreach visits, personnel time for technical and administrative support, travel, and physician opportunity costs. The cost per physician reported by Fretheim et al. was lower than our study possibly because they excluded costs of designing the intervention, which was the key driver of the total costs of ‘MyPractice’.

Our study has certain limitations that must be acknowledged. Data on resources required for development and implementation of the ‘MyPractice’ initiative was collected retrospectively. Personnel time used for each activity may not be accurately reported. Moreover, reduction in prescribing of high-risk antipsychotics could reduce health system cost due to fewer readmissions and outpatient visits. However, we did not account for this potential downstream cost savings due to limited information on long-term effectiveness of A&F interventions. Furthermore, ‘MyPractice’ is a simple audit feedback strategy; therefore, the results from our study may not be generalizable to other audit feedback strategies that may vary in terms of resources required for implementation.

Despite these limitations, our study is one of the few comprehensive economic analyses of an audit and feedback initiative. Findings could help inform the decision to adopt or expand audit and feedback initiatives such as ‘MyPractice’ to address prescribing of high-risk medications in long-term care homes. More studies on the cost of A&F interventions are required as cost data from diverse contexts could highlight A&F design and delivery approaches that can make the intervention more affordable.

## Conclusion

In summary, ‘MyPractice’ is a good return on investment intervention to address prescribing of high-risk medication in long-term care homes. The financial benefits of this and other A&F interventions would depend on how they are designed and delivered. If most intervention costs are either incurred upfront or spread over several years, the A&F interventions may be economically attractive even with a small effect size.

## Supplementary Information

**Additional file 1.**

## Data Availability

Data for cost analysis: The datasets generated during and/or analysed during the current study are available from the corresponding author on reasonable request.

## Abbreviations

A&F: Audit and feedback
FAQs: Frequently asked questions
LTC: Long-term care
ROI: Return on investment

## Footnotes

1 Cost converted from US$ to C$ for comparison purposes.
