# Supplementary material for: Audit feedback interventions to address high-risk prescriptions in long-term care homes: a costing study and return on investment analysis
Source: Implement Sci Commun. 2021 Oct 28;2:125. doi: 10.1186/s43058-021-00225-7 (PMC8554856; doi:10.1186/s43058-021-00225-7)
Supplement: Supplementary file 1 — Additional file 1. [file 43058_2021_225_MOESM1_ESM.docx]

Audit Feedback Interventions to Address High-Risk Prescriptions in Long-Term Care Homes:

A Costing Study and Return on Investment Analysis

# Annexure

Table A1: Distribution of Workload: Percentage of Annual FTE Allocated To Development And Production of ‘MyPractice’ Reports for Antipsychotics Prescribing

| **ROLE** | **ANNUAL FTE** |
| --- | --- |
| **Development Phase** |  |
| Research Analyst | 0.317 |
| HSP Manager | 0.208 |
| Sr. Methodologist | 0.184 |
| QI Manager | 0.138 |
| QI Specialist | 0.102 |
| Administrative Assistant | 0.069 |
| HSP Director | 0.069 |
| Infrastructure Analyst | 0.051 |
| Web/ Application Developer | 0.038 |
| Editor | 0.004 |
| **Implementation Phase** |  |
| Sr. Methodologist | 0.198 |
| Research Analyst | 0.187 |
| Project Coordinator | 0.123 |
| HSP Manager | 0.046 |
| Infrastructure Analyst | 0.044 |
| Administrative Assistant | 0.038 |

FTE, Full Time Equivalent; HSP, Health Service Provider.
